# Multi-omics analysis reveals molecular mechanisms of maize responses to hexavalent chromium toxicity

**DOI:** 10.1186/s12870-026-08643-2

**Published:** 2026-03-26

**Authors:** Huaisheng Zhang, Qianni Xie, Pingxi Wang, Xiangyuan Wu, Xining Jin, Dong Ding, Jiong Wan, Xiaoxiang Zhang

**Affiliations:** 1 School of Agriculture, State Key Laboratory of High-Efficiency Production of Wheat-Maize Double Cropping, Henan Institute of Science and Technology, Xinxiang, 453003 China; 2 State Key Laboratory of High-Efficiency Production of Wheat-Maize Double Cropping, College of Agronomy, Henan Agricultural University, Zhengzhou, 450046 China; 3 Henan Key Laboratory of Tea Plant Biology, College of Tea and Food Science, Xinyang Normal University, Xinyang, Henan 464000 China; 4 Dabie Mountain Laboratory, Xinyang, Henan 464000 China

**Keywords:** Maize, Cr(VI) toxicity, Oxidative stress, Heavy metal tolerance

## Abstract

**Background:**

Hexavalent chromium [Cr(VI)] is a highly toxic heavy metal that adversely affects plant growth and food safety. Here, we investigated the molecular responses of maize seedlings (hybrid Zhengdan958(ZD958) and parental lines Chang7-2(C72) and Zheng58(Z58)) to 20 mg/L Cr(VI) stress using transcriptomic and proteomic analyses.

**Results:**

Cr(VI) treatment induced 1226 commonly upregulated and 655 downregulated genes across all three genotypes, with upregulated genes enriched in stress- and defense-related processes (oxidative stress, xenobiotic response, hormone signaling) and downregulated genes associated with growth and development (cell wall biosynthesis, organ morphogenesis). Proteomic analysis showed similar patterns, highlighting key modules mediating Cr(VI) tolerance. Antioxidant genes (catalase3, glutaredoxin14, acco2, acco5) were significantly upregulated, indicating activation of ROS-scavenging pathways.

**Conclusions:**

These results reveal coordinated transcriptional and proteomic responses that protect maize against Cr(VI)-induced oxidative stress while suppressing growth, providing candidate genes and pathways for breeding maize with enhanced tolerance to heavy metal-contaminated soils.

## Background

The growth and development of plants are inevitably challenged by diverse environmental factors, commonly referred to as stresses, which can be broadly categorized into biotic and abiotic stresses [1–3]. Biotic stresses include damage caused by herbivores [4], insect pests [5], and pathogenic infections [6], whereas abiotic stresses arise from environmental factors such as extreme temperatures [7, 8], drought [9, 10], waterlogging [11], and heavy metal toxicity [12, 13]. In recent decades, rapid industrialization and modern agricultural practices have resulted in the continuous accumulation of heavy metals in soils through the excessive use of pesticides, fertilizers, mining activities, and illegal wastewater discharge [14]. This has disrupted soil ecological balance, caused widespread environmental pollution, and posed serious threats to agricultural productivity and ecological security in China. It is estimated that more than 20 million hectares of arable land in China are contaminated with heavy metals such as Cr, underscoring the urgent need for effective remediation strategies [15, 16].

Heavy metals are generally defined as metallic elements with a density greater than 5 g·cm⁻³, including Cd, Cr, Hg, Pb, Cu, Zn, As, Ag, and Sn [17]. Their toxicity depends largely on the chemical nature of the metal, the duration of exposure, and the plant’s developmental stage. Chromium, the seventh most abundant element in the Earth’s crust, exists in multiple oxidation states, of which trivalent Cr(III) and hexavalent Cr(VI) are the most stable and common [18]. Cr(III) is an essential trace element in humans, functioning as a component of the glucose tolerance factor and contributing to lipid metabolism, while deficiency may impair glucose regulation [19, 20]. In contrast, Cr(VI) is highly toxic, approximately 100 times more harmful than Cr(III), with greater solubility and stability in soil and water, which enhances its bioavailability to plants and leads to water and soil pollution [21, 22]. Numerous studies have reported that Cr(VI) toxicity adversely affects photosynthetic efficiency, induces oxidative stress, and disrupts metabolic homeostasis in crops such as green gram [23], mustard [24], wheat [25], pea [26], and rice [27]. These toxic effects not only reduce plant productivity but also threaten human health as heavy metals enter the food chain through irrigation water and agricultural ecosystems. However, limited studies have investigated the detailed mechanisms of ROS generation, detoxification pathways, and antioxidant defense responses that maintain redox homeostasis in maize.

When plants grow in soils enriched with heavy metals, these elements are absorbed by the roots and accumulate in plant tissues, often leading to visible toxicity symptoms such as stunted growth, chlorosis, leaf wilting, and necrosis [28, 29]. At the cellular and physiological levels, heavy metal stress disrupts membrane structure and function, interferes with nutrient accumulation and hormone regulation, disturbs water homeostasis, and impairs photosynthesis and respiration [30–32]. These processes lead to the excessive accumulation of toxic metabolites such as malondialdehyde (MDA) and hydrogen peroxide (H₂O₂), resulting in oxidative stress and ultimately cell death [33, 34]. To mitigate these adverse effects, plants have evolved a variety of adaptive strategies, including enhanced activities of antioxidant enzymes such as superoxide dismutase (SOD), catalase (CAT), peroxidase (POD), and ascorbate peroxidase (APX) to scavenge excessive reactive oxygen species (ROS), accumulation of osmolytes to stabilize metabolic processes, structural adjustments such as changes in leaf morphology, and up-regulation of stress-responsive genes to improve tolerance [35, 36].

In this study, we evaluate the effects of increasing Cr(VI) exposure on maize seedling growth, and characterize the associated transcriptomic and proteomic changes, in order to gain a better understanding of Cr(VI) tolerance mechanisms in maize. These findings will not only provide new insights into the defense strategies of maize under heavy metal stress but also offer a theoretical basis for developing Cr(VI)-tolerant crop varieties and guiding the ecological remediation of Cr(VI)-contaminated soils.

## Methods

### Plant material and seed germinating condition

An elite maize hybrid, ZD958, and its parental inbred lines, Z58 and C72, were selected as experimental materials. Fifty healthy seeds of uniform size, free from disease and insect damage, were chosen from each genotype. The selected seeds were surface-sterilized by immersion in 0.1% HgCl₂ solution for 20 min, followed by 8–10 rinses with sterile water. Sterilized fine sand (autoclaved) was filled into small plastic cups with drainage holes at the bottom, and four seeds were sown per cup at a depth of 3–4 cm. After sowing, sufficient water was added to maintain sand moisture for seed germination. The cups were then placed in trays, clearly labeled with genotype and sowing date, and transferred to a controlled growth chamber maintained at 25 °C, 75% relative humidity, with a 14 h light/10 h dark photoperiod. During germination, seedling emergence was monitored daily, and water was supplemented as needed. After 7–10 days of growth, when seedlings reached the three-leaf stage, they were transferred to hydroponic culture boxes for subsequent experiments.

### Cr(VI) treatment and sample collection

In a preliminary experiment, six concentrations of K₂CrO₄ (0, 5, 10, 20, 50, and 100 mg L⁻¹) were tested. No visible stress symptoms were observed at 0, 5, and 10 mg L⁻¹ after 10 days, while severe leaf wilting and growth inhibition occurred at 50 and 100 mg L⁻¹. Therefore, 20 mg L⁻¹ K₂CrO₄ was selected for subsequent experiments. Maize seedlings at the three-leaf stage were transferred to hydroponic boxes containing modified Hoagland nutrient solution (NS10205 500×). Seedlings with uniform growth were carefully cleaned to remove residual endosperm tissue, rinsed with deionized water, and fixed with foam supports. Seedlings were then divided into two groups: (i) control (0 mg L⁻¹ Cr), and (ii) treatment (20 mg L⁻¹ Cr). Seedlings were grown under the same controlled conditions, with nutrient solution replaced every three days to maintain sufficient oxygen supply for root development. After 10 days of treatment, seedlings were harvested. Excess water was removed from roots with absorbent paper, and root tissues were excised, immediately wrapped in foil, snap-frozen in liquid nitrogen, and stored on dry ice. Samples were then sent to LC-Bio (Hangzhou, China) for transcriptome and proteome sequencing. Three biological replicates and three technical replicates were performed for each treatment.

### Analysis of RNA-seq

Raw sequencing data were processed with FASTP to remove adapter sequences and low-quality reads [37]. Clean reads were aligned to the maize B73 reference genome using HISAT2 [38]. Gene expression levels were quantified with FeatureCounts [39], and differential expression analysis was conducted using DESeq2 [40, 41], with thresholds set at |fold change| ≥ 2 and false discovery rate (FDR) < 0.05. Gene Ontology (GO) annotations were obtained based on the longest protein sequences via the eggNOG-mapper platform [42]. Functional enrichment analyses for GO terms were performed using the clusterProfiler package in R [43].

### Protein extraction and data analysis

Root samples were ground into fine powder in liquid nitrogen and proteins were precipitated with 10% (w/v) trichloroacetic acid in cold acetone (− 20 °C). After centrifugation at 20,000 × g for 30 min at 4 °C, the pellet was washed three times with 80% cold acetone and subsequently dissolved in 6 M guanidine hydrochloride prepared in 50 mM Tris–HCl (pH 8.0). The solution was clarified by ultracentrifugation at 100,000 × g for 1 h. For reduction and alkylation, 2 mg of protein from each sample was incubated with 20 mM dithiothreitol at 60 °C for 30 min, followed by treatment with 40 mM iodoacetamide for 1 h in the dark at room temperature. The samples were dialyzed sequentially against 2 M urea in 50 mM NH₄HCO₃ and then against 50 mM NH₄HCO₃ to remove excess reagents. Protein concentrations were determined using the Bradford assay according to the manufacturer’s instructions. Differentially expressed proteins were identified based on a fold change ≥ 1.5 or ≤ 0.67 with a P value ≤ 0.05 (Student’s *t*-test).

### Weighted gene co-expression network analysis (WGCNA)

Corrected TPM values were used for WGCNA [44]. Genes with average TPM < 1 across all 18 samples were filtered out, and the remaining genes were subjected to network construction. The soft-thresholding power (β) was set to 11. Modules were identified using the dynamic tree cut algorithm with a minimum module size of 50 genes, and similar modules were merged at a cut height of 0.15. Modules with a *p* < 0.05 in the module–trait correlation analysis were defined as significantly associated with traits.

### Gene expression verification

To validate RNA-seq results, several key genes were selected for RT-qPCR analysis. Primer sequences are provided in Table S1. Total RNA was extracted from the same samples used for transcriptome and proteome analyses using TRIzol reagent (Invitrogen), following the manufacturer’s instructions. RNA was treated with DNase I to remove genomic DNA, and reverse-transcribed into cDNA using the SYBR Premix Ex Taq II (Tli RNaseH Plus) kit. RT-qPCR was performed on a CFX96 RT-qPCR Detection System. The housekeeping gene *ZmActin1* was used as an internal reference, and relative expression levels were calculated using the 2^−ΔΔCt^ method.

## Results

### Cr(VI) exposure causes severe phytotoxic effects in maize seedlings

**Fig. 1:**
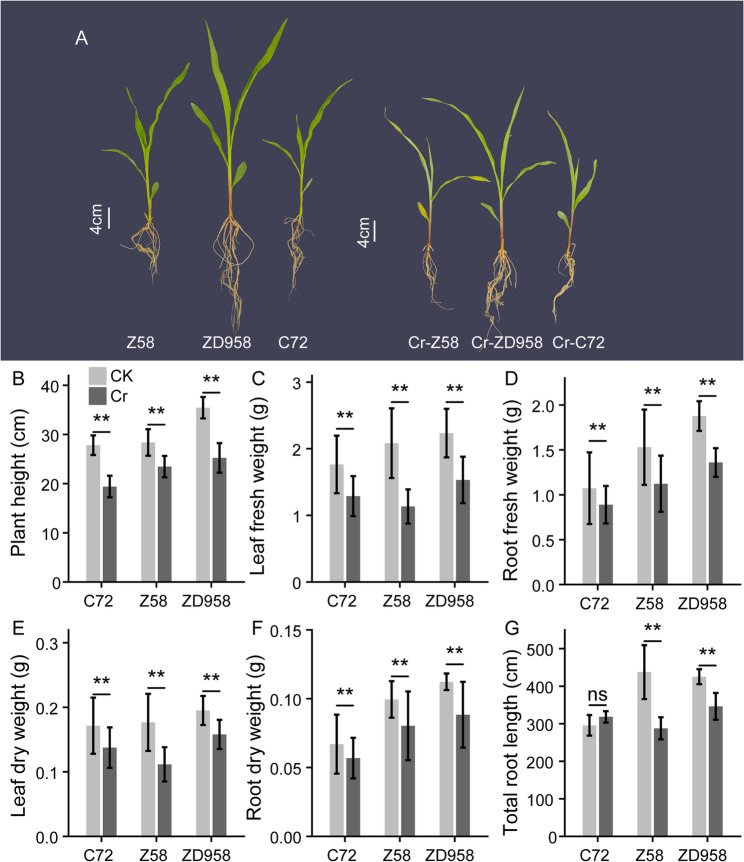
Responses of maize seedlings to Cr(VI) stress. (**A**) Phenotypic comparison between control and Cr(VI)-treated seedlings in three genotypes (Zheng58, Chang7-2, and Zhengdan958); scale bar = 10 cm. Phenotypic differences for (**B**) Plant height, (**C**) Leaf fresh weight, (**D**) Root fresh weight, (**E**) Leaf dry weight, (**F**) Root dry weight, (**G**) Root length, Data are presented as mean ± SD. Statistical significance was determined using a two-tailed Student’s *t*-test, sample number *n* = 5, ** indicates *P* < 0.01, ‘ns’ indicates *P* > 0.05

Cr(VI) exposure exerted significant inhibitory effects on the growth of maize seedlings (Fig. 1A). Compared with the control, plant height was markedly reduced under Cr(VI) treatment across all three genotypes (Fig. 1B). In addition, both root and shoot biomass, measured as fresh and dry weights, were significantly reduced in Cr(VI)-treated seedlings of all genotypes (Fig. 1C-F). Furthermore, total root length was significantly reduced in Cr(VI)-treated Z58 and ZD958 compared with their respective controls, whereas no significant difference was observed in C72 (Fig. 1G). These results indicate that Cr(VI) exposure causes severe phytotoxicity, leading to substantial inhibition of maize seedling growth.

### Chromium-responsive genes identified by RNA-seq

**Fig. 2:**
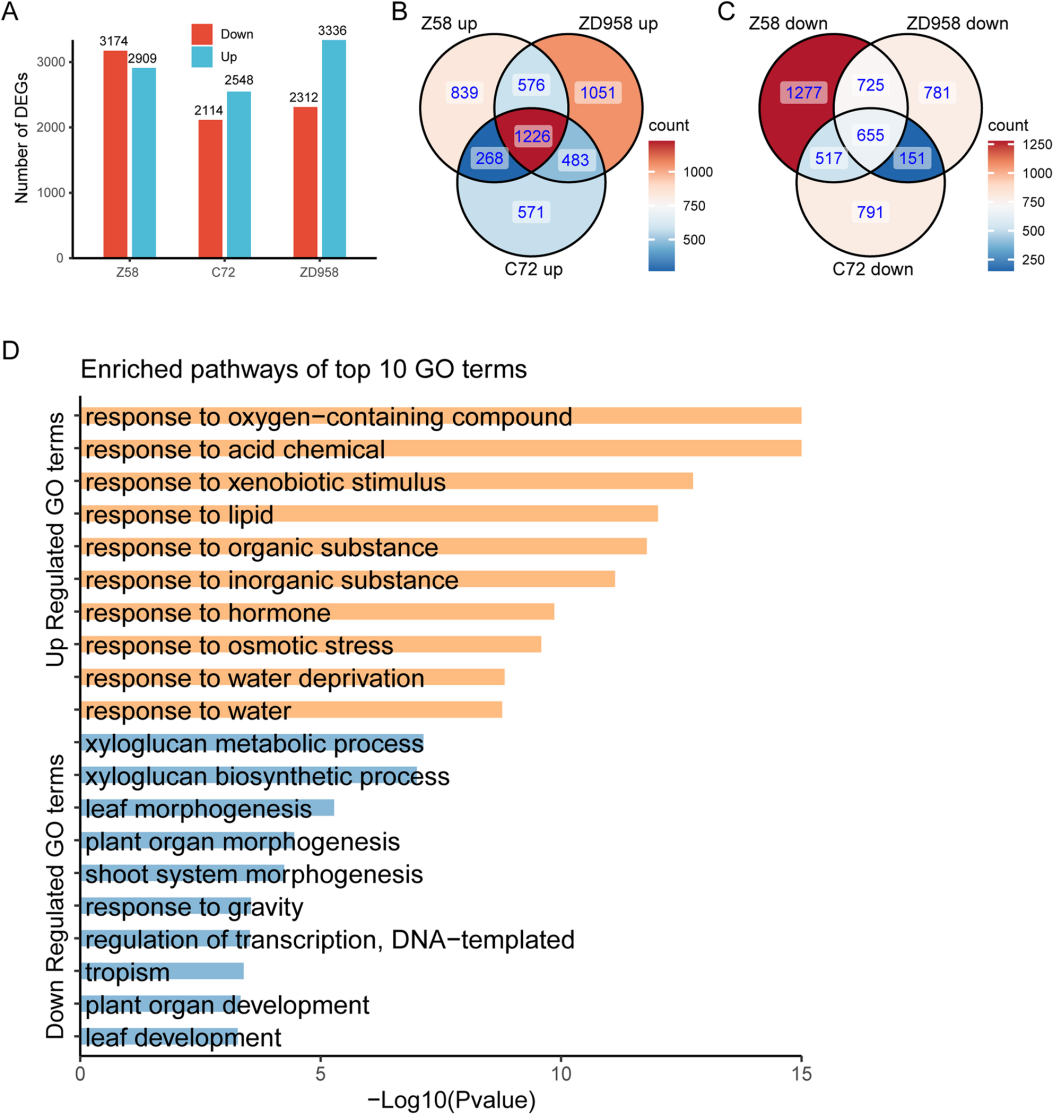
Differentially expressed genes and their GO enrichment analysis under Cr(VI) stress. **A** Numbers of DEGs identified in Cr(VI)-treated roots of Zheng58, Chang7-2, and Zhengdan958. Red bars represent downregulated genes, while blue bars represent upregulated genes. **B** Venn diagram of upregulated genes in the three genotypes. The overlap of 1226 genes indicate the common upregulated DEGs across all genotypes in Cr(VI)-treated roots. **C** Venn diagram of downregulated genes in the three genotypes. The overlap of 655 genes indicates the common downregulated DEGs across all genotypes in Cr(VI)-treated roots. **D** GO enrichment analysis showing the top 10 enriched pathways for the 1226 common upregulated DEGs and 655 common downregulated DEGs, respectively

To explore the transcriptional response of maize roots to Cr(VI) stress, roots of C72, Z58, and ZD958 under control and Cr(VI) treatment were collected for transcriptome sequencing. Differential expression analysis identified 6083 differentially expressed genes (DEGs) in Cr(VI)-treated Zheng58 roots, including 2909 upregulated and 3174 downregulated genes. In Chang7-2, 4662 DEGs were detected (2548 upregulated and 2114 downregulated), whereas in Zhengdan958, 5648 DEGs were identified (3336 upregulated and 2312 downregulated) (Fig. 2A). Among these DEGs, 1226 genes were commonly upregulated and 655 genes were commonly downregulated across the three genotypes (Fig. 2B, C). GO enrichment analysis revealed that the 1226 commonly upregulated genes were enriched in biological processes such as response to oxygen-containing compound, response to acid chemical, response to xenobiotic stimulus, response to hormone, response to osmotic stress, and response to water deprivation. In contrast, the 655 commonly downregulated genes were mainly enriched in xyloglucan metabolic process, plant organ morphogenesis, response to gravity, regulation of transcription (DNA-templated), and plant organ development (Fig. 2D, Supplemental Table 1). Since transcription factors (TFs) are known to play crucial roles in stress-induced transcriptional reprogramming, further analysis showed that 736 TF-encoding genes were differentially expressed, among which MYB (77), bHLH (70), ERF (64), NAC (63) and WRKY (55) families were the most prominent (Supplemental Table 2).

### Effect of Cr (VI) on protein accumulation

**Fig. 3:**
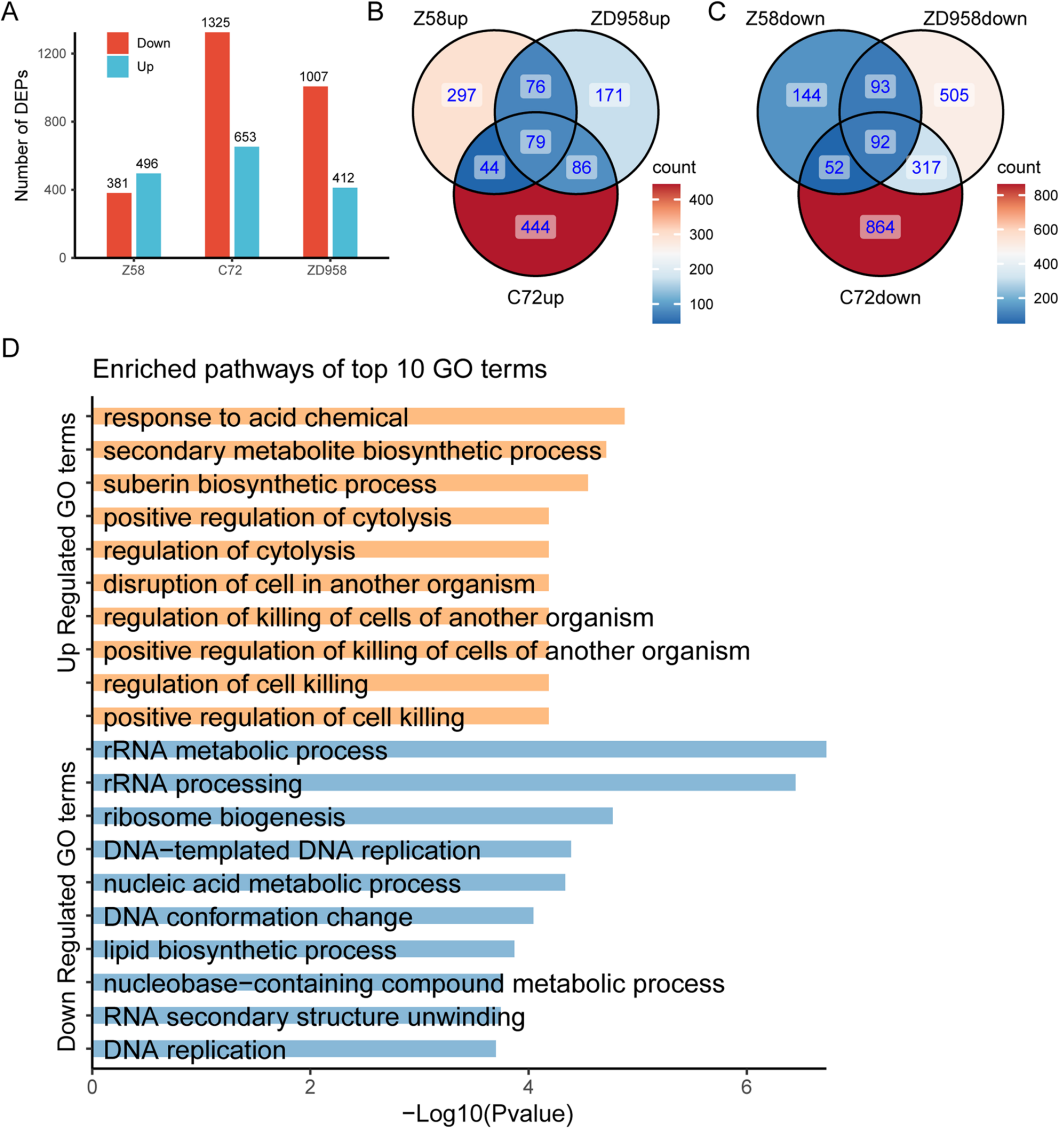
Differentially expressed proteins and their GO enrichment analysis under Cr(VI) stress. **A** Numbers of DEPs identified in Cr(VI)-treated roots of Zheng58, Chang7-2, and Zhengdan958. Red bars represent downregulated proteins, while blue bars represent upregulated proteins. **B** Venn diagram of upregulated proteins in the three genotypes. The overlap of 79 proteins indicates the common upregulated DEPs across all genotypes in Cr(VI)-treated roots. **C** Venn diagram of downregulated proteins in the three genotypes. The overlap of 92 proteins indicates the common downregulated DEPs across all genotypes in Cr(VI)-treated roots. **D** GO enrichment analysis showing the top 10 enriched pathways for the union of upregulated DEPs and the union of downregulated DEPs across the three genotypes

To examine proteomic changes under Cr(VI) stress, the same root samples used for transcriptome analysis were subjected to quantitative proteomics. In Cr(VI)-treated Z58 roots, 877 DEPs were identified (496 upregulated, 381 downregulated). In C72, 1978 DEPs were detected (653 upregulated, 1325 downregulated), while in ZD958, 1419 DEPs were identified (412 upregulated, 1007 downregulated) (Fig. 3A). A total of 79 proteins were commonly upregulated and 92 proteins were commonly downregulated across the three genotypes (Fig. 3B, C). Given the relatively small number of common DEPs, the union of DEPs across the three genotypes was used for GO enrichment analysis. The upregulated proteins were mainly enriched in response to acid chemical, suberin biosynthetic process, positive regulation of cytolysis, and regulation of killing of cells of another organism. Conversely, the downregulated proteins were predominantly enriched in rRNA metabolic process, ribosome biogenesis, and DNA-templated DNA replication (Fig. 3D, Supplemental Table 3). Notably, the upregulated categories were largely associated with stress responses and irreversible programmed cell death triggered by prolonged exposure, whereas the downregulated categories were related to essential cellular processes required for survival.

### Weighted gene co-expression network analysis

**Fig. 4:**
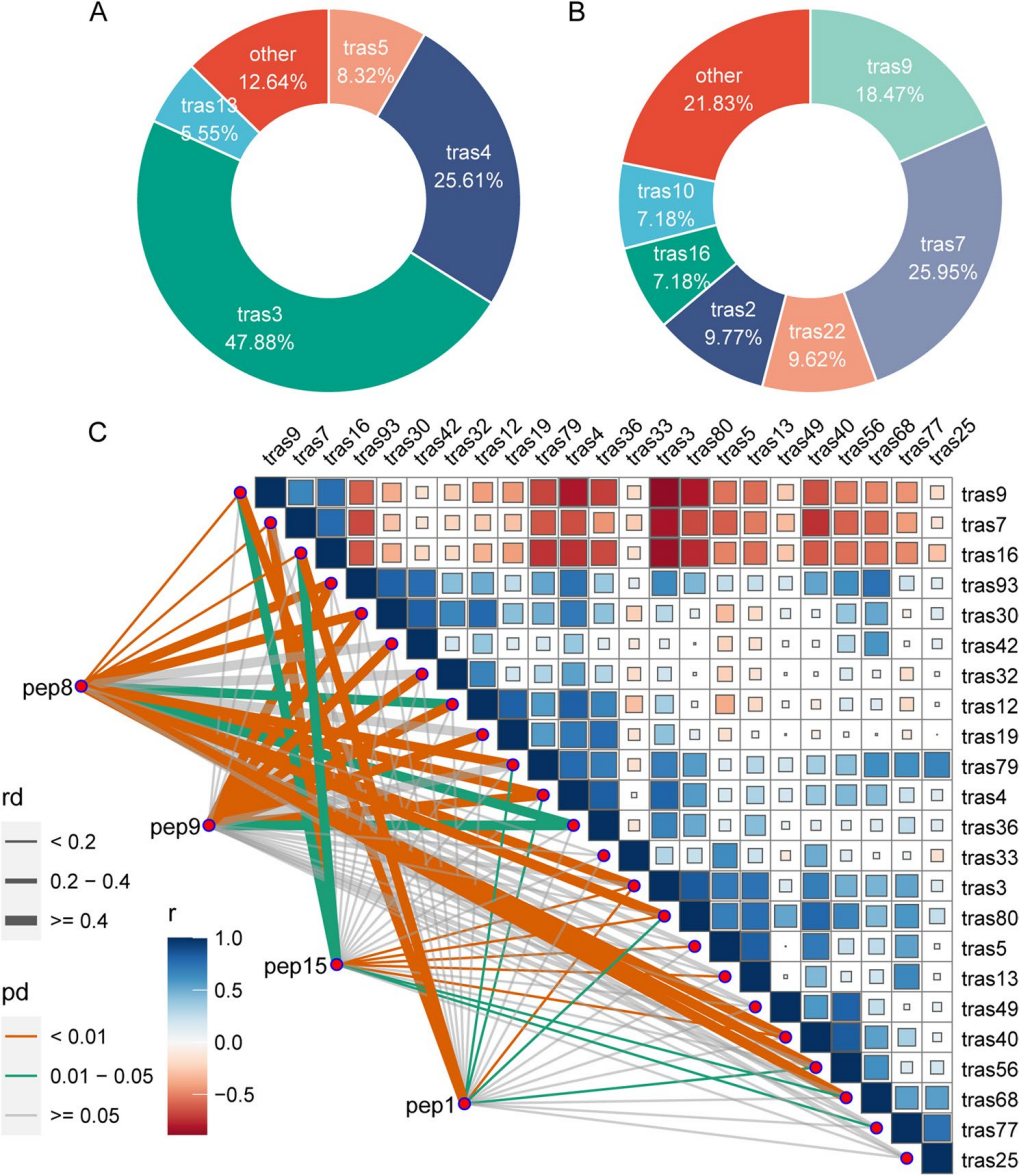
WGCNA-based module construction and correlation analysis between transcriptomic and proteomic modules. **A** Distribution of the 1226 common upregulated DEGs among transcriptome-based modules. **B** Distribution of the 655 common downregulated DEGs among transcriptome-based modules. **C** Correlation between modules defined by transcriptomic data and those defined by proteomic data. *r* indicates the correlation coefficient between transcriptomic modules, *pd* represents the p-value assessing the significance of correlations between transcriptomic and proteomic modules, and *rd* denotes the correlation coefficient range

To identify co-regulated genes and proteins in response to Cr(VI) stress, weighted gene co-expression network analysis (WGCNA) was performed separately on transcriptome and proteome datasets. After filtering out lowly expressed genes, 33,151 genes were grouped into 105 modules. Interestingly, the 1226 common upregulated genes were mainly clustered in modules trans3 (47.88%) and trans4 (25.16%) (Fig. 4A). In contrast, the 655 common downregulated genes were more dispersed, with relatively higher proportions in modules trans7 (25.95%) and trans9 (18.47%) (Fig. 4B). For the proteomic dataset, 10,467 proteins were classified into 64 modules. Correlation analysis revealed that two protein modules, pep8 and pep9, were strongly associated with transcriptomic modules trans3 and trans4 (Fig. 4C). Importantly, 48 (61%) of the 79 commonly upregulated proteins were located within pep8 and pep9. Collectively, these results suggest that genes and proteins grouped in modules trans3, trans4, pep8, and pep9 likely represent key regulators in the maize response to Cr(VI) stress.

### Validation of DEGs by quantitative real-time PCR

**Fig. 5:**
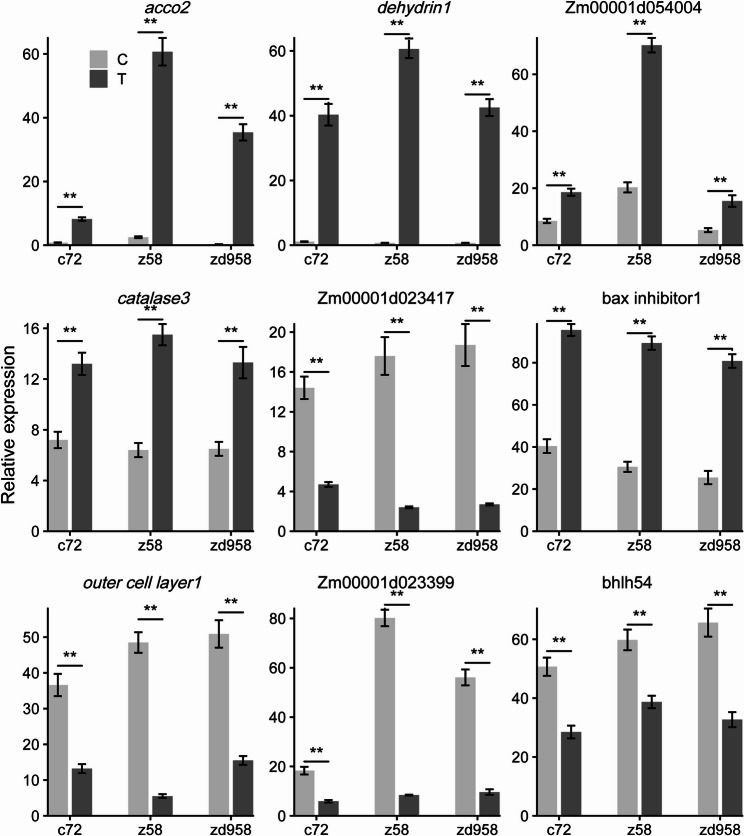
Validation of relative expression level by qRT‒PCR. Relative expression levels of several selected DEGs were validated using qRT‒PCR analysis. ** indicates *P* < 0.01

To validate the reliability of RNA-seq results, several DEGs associated with Cr(VI) response were selected for qRT-PCR analysis. These genes represent different functional categories identified in the transcriptomic analysis, including oxidative stress response, cell wall organization, and transcriptional regulation. For example, zeaxanthin epoxidase2, dehydrin1, bax inhibitor1, catalase3, 1-aminocyclopropane-1-carboxylate oxidase5, and 1-aminocyclopropane-1-carboxylate oxidase2 were enriched in the response to oxygen-containing compound category. Outer cell layer1 was associated with cell wall organization or biogenesis, while Aux/IAA transcription factor 22 and bHLH transcription factor 54 were related to regulation of transcription (DNA-templated). The qRT-PCR results confirmed that the expression trends of these genes were consistent with the RNA-seq data (Fig. 5).

## Discussion

### Toxic effects of Cr(VI) and maize responses

Maize is exposed to a variety of biotic and abiotic stresses throughout its life cycle, ranging from drought, salinity, extreme temperatures, and pathogen infection to heavy metal toxicity [45, 46]. Transcriptional reprogramming during abiotic stress is crucial for stress tolerance. For instance, ZmHSF08, encoding a UDP-glycosyltransferase, is induced under heat stress and contributes to heat tolerance by regulating ROS homeostasis [47, 48], while ZmWRKY64 enhances cadmium tolerance by modulating metal uptake, transport, and ROS scavenging [49]. Therefore, we examined the transcriptomic and proteomic responses of maize seedlings to Cr(VI) stress using the elite hybrid ZD958 and its parental lines C72 and Z58.

Our results showed that upregulated genes were significantly enriched in GO terms associated with stress and defense responses, including response to oxygen-containing compound, response to acid chemical, response to xenobiotic stimulus, response to hormone, and response to water deprivation. This indicates that Cr(VI) stress activates pathways related to oxidative stress, chemical detoxification, hormone signaling, and osmotic adjustment. In contrast, downregulated genes were enriched in growth and developmental processes, such as xyloglucan biosynthetic process, plant organ morphogenesis, shoot system morphogenesis, and plant organ development. This suggests that cell wall biosynthesis, organ development, and transcriptional regulation are suppressed under prolonged Cr(VI) exposure.

**Fig. 6:**
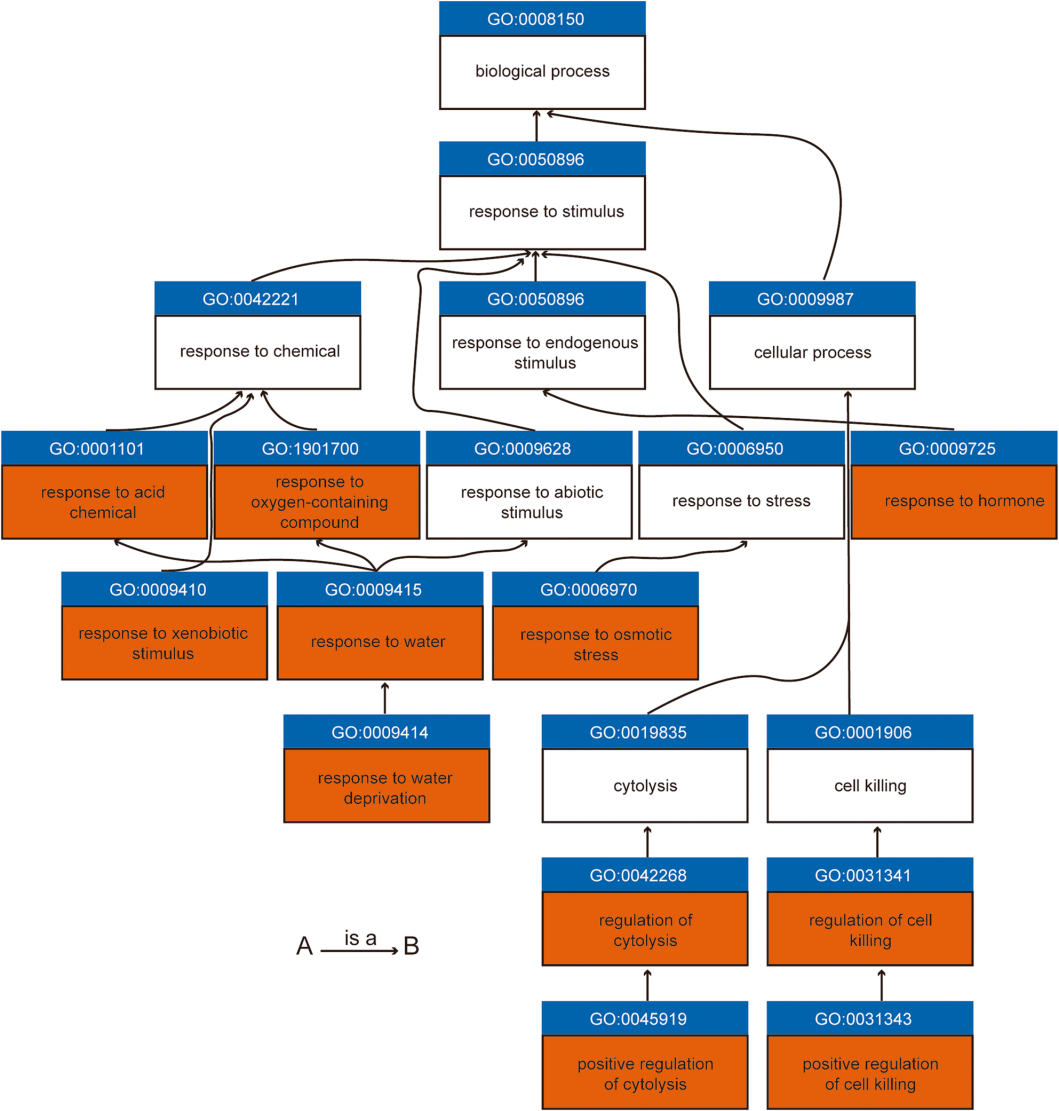
Workflow of the main upregulated GO terms under Cr(VI) stress. The diagram illustrates the union of upregulated GO terms identified from both transcriptomic and proteomic analyses. Brown highlighted boxes indicate GO terms that are significantly enriched in either upregulated genes or upregulated proteins

At the proteomic level, upregulated proteins were enriched in processes related to cell death (e.g., positive regulation of cytolysis, positive regulation of cell killing) (Fig. 6), while downregulated proteins were associated with fundamental cellular processes (e.g., rRNA processing, ribosome biogenesis, and DNA replication). Together, these findings imply that when maize seedlings are subjected to prolonged Cr(VI) stress, initial defense mechanisms such as oxidative stress responses and osmotic adjustment may become insufficient, ultimately leading to programmed cell death. Overall, these observations are consistent with typical plant responses to heavy metal stress, where defense and detoxification mechanisms are activated, often accompanied by growth inhibition [50, 51].

### Biological significance and mechanistic insights

Our integrated transcriptomic–proteomic analysis provides a comprehensive view of maize seedling responses to Cr(VI) stress that aligns well with established heavy-metal defense strategies. It is well recognized that plants limit heavy-metal uptake and reinforce cell walls and vacuoles to sequester toxic ions. Consistent with this, we observed increased expression of cell-wall biosynthesis genes (e.g. *cellulose synthase-like protein D3* and *microtubule-associated protein*) and upregulation of thiol-based detoxification enzymes. Likewise, antioxidant enzymes (e.g. *peroxidase23* and *antioxidant enzyme catalase3*) were more abundant, reflecting activation of the known antioxidative defense network. These coordinated changes underscore that cell-wall immobilization and redox homeostasis are core aspects of maize tolerance to Cr(VI) stress.

These observations are consistent with previous studies on chromium toxicity in maize. For example, Yang and colleagues showed that exogenous H₂S under Cr stress upregulated pectin biosynthesis and PME activity, causing more Cr to be retained in the cell wall, and boosted glutathione and phytochelatin levels for vacuolar detoxification [52]. Similarly, several genes involved in cell-wall polysaccharide synthesis and glutathione metabolism were strongly induced in our dataset, suggesting that maize may activate comparable detoxification pathways even in the absence of exogenous H₂S. In addition, Adhikari et al. demonstrated that Cr(VI) exposure causes severe oxidative stress in maize seedlings, including reductions in plant height and biomass together with strong accumulation of reactive oxygen species [33]. In agreement with these physiological observations, our multi-omics data revealed increased abundance of antioxidative enzymes and stress-responsive proteins, supporting the idea that oxidative stress is a central component of maize responses to Cr(VI).

Taken together, these comparisons indicate that our results are broadly consistent with previously reported mechanisms of chromium tolerance while also providing additional insights from an integrated multi-omics perspective. By combining transcriptomic and proteomic analyses, we were able to identify genes and proteins that showed coordinated regulation under Cr(VI) stress. These commonly regulated components are likely to represent core elements of the maize response to chromium toxicity. Compared with single-omics approaches, this integrative strategy helps to narrow the range of candidate genes and provides a more robust framework for identifying key regulators involved in stress adaptation. Overall, this study provides new insights into the molecular mechanisms underlying maize adaptation to Cr(VI) stress and highlights potential candidate genes for developing maize varieties with enhanced tolerance to heavy metal toxicity, thereby offering theoretical guidance and genetic resources for breeding programs aimed at improving crop resilience in contaminated soils.

## Data Availability

The raw sequencing data reported in this study are archived at the National Genomics Data Center under BioProject accession number PRJNA1347427. Additionally, supporting data for the findings presented in this study can be found either within the manuscript or in the supplementary files.

## Abbreviations

Z58: Zheng58
C72: Chang7-2
ZD958: Zhengdan958
Cr: Chromium
RT-qPCR: Real-time quantitative PCR
TFs: Transcription factors
MDA: Malondialdehyde
H_2_O_2_: Hydrogen peroxide
SOD: Superoxide dismutase
CAT: Catalase
POD: Peroxidase
APX: Ascorbate peroxidase
ROS: Reactive oxygen species
GO: Gene Ontology
DEGs: Differentially Expressed Genes
WGCNA: Weighted gene co-expression network analysis

## References

[1] Zhu JK. Abiotic Stress Signaling and Responses in Plants. Cell. 2016;167(2):313–24. 27716505 10.1016/j.cell.2016.08.029 PMC5104190

[2] Zhao D, Wang H, Chen S, Yu D, Reiter RJ. Phytomelatonin: An Emerging Regulator of Plant Biotic Stress Resistance. Trends Plant Sci. 2021;26(1):70–82. 32896490 10.1016/j.tplants.2020.08.009

[3] Ge L, Pan F, Jia M, Pott DM, He H, Shan H, Lozano-Durán R, Wang A, Zhou X, Li F. RNA modifications in plant biotic interactions. Plant Commun. 2025;6(2):101232. 39722456 10.1016/j.xplc.2024.101232 PMC11897454

[4] Zhou W, Lozano-Torres JL, Blilou I, Zhang X, Zhai Q, Smant G, Li C, Scheres B. A Jasmonate Signaling Network Activates Root Stem Cells and Promotes Regeneration. Cell. 2019;177(4):942–e956914. 30955889 10.1016/j.cell.2019.03.006

[5] Islam W, Tayyab M, Khalil F, Hua Z, Huang Z, Chen HYH. Silicon-mediated plant defense against pathogens and insect pests. Pestic Biochem Physiol. 2020;168:104641. 32711774 10.1016/j.pestbp.2020.104641

[6] Bai Y, Kissoudis C, Yan Z, Visser RGF, van der Linden G. Plant behaviour under combined stress: tomato responses to combined salinity and pathogen stress. Plant J. 2018;93(4):781–93. 29237240 10.1111/tpj.13800

[7] Ding Y, Shi Y, Yang S. Molecular Regulation of Plant Responses to Environmental Temperatures. Mol Plant. 2020;13(4):544–64. 32068158 10.1016/j.molp.2020.02.004

[8] Kidokoro S, Shinozaki K, Yamaguchi-Shinozaki K. Transcriptional regulatory network of plant cold-stress responses. Trends Plant Sci. 2022;27(9):922–35. 35210165 10.1016/j.tplants.2022.01.008

[9] Chen L, Yang H, Fang Y, Guo W, Chen H, Zhang X, Dai W, Chen S, Hao Q, Yuan S, et al. Overexpression of GmMYB14 improves high-density yield and drought tolerance of soybean through regulating plant architecture mediated by the brassinosteroid pathway. Plant Biotechnol J. 2021;19(4):702–16. 33098207 10.1111/pbi.13496 PMC8051608

[10] Wang X, Wang H, Liu S, Ferjani A, Li J, Yan J, Yang X, Qin F. Genetic variation in ZmVPP1 contributes to drought tolerance in maize seedlings. Nat Genet. 2016;48(10):1233–41. 27526320 10.1038/ng.3636

[11] Yu F, Liang K, Zhang Z, Du D, Zhang X, Zhao H, Ui Haq B, Qiu F. Dissecting the genetic architecture of waterlogging stress-related traits uncovers a key waterlogging tolerance gene in maize. Theor Appl Genet. 2018;131(11):2299–310. 30062652 10.1007/s00122-018-3152-0

[12] Hou F, Liang Y, Sang M, Zhao G, Song J, Liu P, Zou C, Chen Z, Ma L, Shen Y. Complex regulatory network of ZmbZIP54-mediated Pb tolerance in maize. Plant Physiol Biochem. 2025;224:109945. 10.1016/j.plaphy.2025.109945 40279841

[13] Wang K, Wu Z, Zhang M, Lu X, Lai J, Zhang M, Wang Y. Metal ion transport in maize: survival in a variable stress environment. J Genet Genomics. 2025;52(3):297–306. 39824435 10.1016/j.jgg.2025.01.005

[14] Sun L, Zhang X, Ouyang W, Yang E, Cao Y, Sun R. Lowered Cd toxicity, uptake and expression of metal transporter genes in maize plant by ACC deaminase-producing bacteria Achromobacter sp. J Hazard Mater. 2022;423(Pt A):127036. 34481390 10.1016/j.jhazmat.2021.127036

[15] Yuan X, Xue N, Han Z. A meta-analysis of heavy metals pollution in farmland and urban soils in China over the past 20 years. J Environ Sci. 2021;101:217–26. 10.1016/j.jes.2020.08.013 33334517

[16] Chen D, Hu W. Temporal and spatial effects of heavy metal-contaminated cultivated land treatment on agricultural development resilience. Land. 2023;12(5):945.

[17] Arora NK, Chauhan R. Heavy metal toxicity and sustainable interventions for their decontamination. Environ Sustain. 2021;4(1):1–3.

[18] He L, Wang M, Zhang G, Qiu G, Cai D, Wu Z, Zhang X. Remediation of Cr(VI) contaminated soil using long-duration sodium thiosulfate supported by micro-nano networks. J Hazard Mater. 2015;294:64–9. 25855614 10.1016/j.jhazmat.2015.03.052

[19] Eastmond DA, Macgregor JT, Slesinski RS. Trivalent chromium: assessing the genotoxic risk of an essential trace element and widely used human and animal nutritional supplement. Crit Rev Toxicol. 2008;38(3):173–90. 18324515 10.1080/10408440701845401

[20] Markiewicz B, Komorowicz I, Sajnóg A, Belter M, Barałkiewicz D. Chromium and its speciation in water samples by HPLC/ICP-MS–technique establishing metrological traceability: a review since 2000. Talanta. 2015;132:814–28. 25476383 10.1016/j.talanta.2014.10.002

[21] Xia X, Wu S, Zhou Z, Wang G. Microbial Cd(II) and Cr(VI) resistance mechanisms and application in bioremediation. J Hazard Mater. 2021;401:123685. 33113721 10.1016/j.jhazmat.2020.123685

[22] Shanker AK, Cervantes C, Loza-Tavera H, Avudainayagam S. Chromium toxicity in plants. Environ Int. 2005;31(5):739–53. 15878200 10.1016/j.envint.2005.02.003

[23] Ali HH, Ilyas M, Zaheer MS, Hameed A, Ikram K, Khan WD, Iqbal R, Awan TH, Rizwan M, Mustafa AE-ZM. Alleviation of chromium toxicity in mung bean (Vigna radiata L.) using salicylic acid and Azospirillum brasilense. BMC Plant Biol. 2023;23(1):535. 37919670 10.1186/s12870-023-04528-w PMC10623693

[24] Asgher M, Sehar Z, Fatma M, Hanief M, Shah AA, Khan NA. Ethylene and spermine attenuate chromium-inhibited photosynthetic functions by improving nitrogen and sulfur assimilation and antioxidant system in mustard. Plant Stress. 2023;9:100196.

[25] Yang Y, Peng Y, Ma Y, Chen G, Li F, Liu T. Effects of aging and reduction processes on Cr toxicity to wheat root elongation in Cr (VI) spiked soils. Environ Pollut. 2022;296:118784. 34979171 10.1016/j.envpol.2021.118784

[26] Wu H-s, Yang G-y, Ding J, Tian W, Wu Y-c, Di M-c, Duan Y-j, Li Y-h, Liu Z. Feng Y-c: Effects of chromium and lead mixture on pea’s growth, ultrastructure, translocation and their accumulation in organs. Sci Hort. 2023;314:111940.

[27] Riaz A, Qin Y, Zheng Q, Chen X, Jiang W, Riaz B, Xiao N, Wu X, Qiu X, Xu J, et al. Cr(VI) behaves differently than Cr(III) in the uptake, translocation and detoxification in rice roots. Sci Total Environ. 2024;948:174736. 39029762 10.1016/j.scitotenv.2024.174736

[28] Wang P, Li M, Ma X, Zhao B, Jin X, Chen S, Zhang X, Wu X, Zhang H. Integrated physiological, transcriptomic and metabolomic analysis revealed heterosis for cadmium tolerance in maize. Plant Physiol Biochem. 2025;228:110265. 10.1016/j.plaphy.2025.110265 40695211

[29] Altaf MA, Hao Y, Shu H, Mumtaz MA, Cheng S, Alyemeni MN, Ahmad P, Wang Z. Melatonin enhanced the heavy metal-stress tolerance of pepper by mitigating the oxidative damage and reducing the heavy metal accumulation. J Hazard Mater. 2023;454:131468. 37146338 10.1016/j.jhazmat.2023.131468

[30] Singh S, Kumar V, Parihar P, Dhanjal DS, Singh R, Ramamurthy PC, Prasad R, Singh J. Differential regulation of drought stress by biological membrane transporters and channels. Plant Cell Rep. 2021;40(8):1565–83. 34132878 10.1007/s00299-021-02730-4

[31] Wang R, Fei Y, Pan Y, Zhou P, Adegoke JO, Shen R, Lan P. IMA peptides function in iron homeostasis and cadmium resistance. Plant Sci. 2023;336:111868. 37722507 10.1016/j.plantsci.2023.111868

[32] Sagardoy R, Vázquez S, Florez-Sarasa ID, Albacete A, Ribas-Carbó M, Flexas J, Abadía J, Morales F. Stomatal and mesophyll conductances to CO2 are the main limitations to photosynthesis in sugar beet (Beta vulgaris) plants grown with excess zinc. New Phytol. 2010;187(1):145–58. 20374501 10.1111/j.1469-8137.2010.03241.x

[33] Adhikari A, Adhikari S, Ghosh S, Azahar I, Shaw AK, Roy D, Roy S, Saha S, Hossain Z. Imbalance of redox homeostasis and antioxidant defense status in maize under chromium (VI) stress. Environ Exp Bot. 2020;169:103873.

[34] Noor I, Sohail H, Sun J, Nawaz MA, Li G, Hasanuzzaman M, Liu J. Heavy metal and metalloid toxicity in horticultural plants: Tolerance mechanism and remediation strategies. Chemosphere. 2022;303(Pt 3):135196. 35659937 10.1016/j.chemosphere.2022.135196

[35] Zhang FQ, Wang YS, Lou ZP, Dong JD. Effect of heavy metal stress on antioxidative enzymes and lipid peroxidation in leaves and roots of two mangrove plant seedlings (Kandelia candel and Bruguiera gymnorrhiza). Chemosphere. 2007;67(1):44–50. 17123580 10.1016/j.chemosphere.2006.10.007

[36] Chebbi L, Boughattas I, Helaoui S, Mkhinini M, Jabnouni H, Ben Fadhl E, Alphonse V, Livet A, Giusti-Miller S, Banni M, et al. Environmental microplastic interact with heavy metal in polluted soil from mine site in the North of Tunisia: Effects on heavy metal accumulation, growth, photosynthetic activities, and biochemical responses of alfalfa plants (Medicago saliva L). Chemosphere. 2024;362:142521. 38857630 10.1016/j.chemosphere.2024.142521

[37] Chen S. Ultrafast one-pass FASTQ data preprocessing, quality control, and deduplication using fastp. Imeta. 2023;2(2):e107. 38868435 10.1002/imt2.107 PMC10989850

[38] Kim D, Paggi JM, Park C, Bennett C, Salzberg SL. Graph-based genome alignment and genotyping with HISAT2 and HISAT-genotype. Nat Biotechnol. 2019;37(8):907–15. 31375807 10.1038/s41587-019-0201-4 PMC7605509

[39] Liao Y, Smyth GK, Shi W. featureCounts: an efficient general purpose program for assigning sequence reads to genomic features. Bioinformatics. 2014;30(7):923–30. 24227677 10.1093/bioinformatics/btt656

[40] Love MI, Huber W, Anders S. Moderated estimation of fold change and dispersion for RNA-seq data with DESeq2. Genome Biol. 2014;15(12):550. 25516281 10.1186/s13059-014-0550-8 PMC4302049

[41] Liu S, Wang Z, Zhu R, Wang F, Cheng Y, Liu Y. Three Differential Expression Analysis Methods for RNA Sequencing: limma, EdgeR, DESeq2. JoVE. 2021;(175):e62528. 10.3791/62528 34605806

[42] Cantalapiedra CP, Hernández-Plaza A, Letunic I, Bork P, Huerta-Cepas J. eggNOG-mapper v2: Functional Annotation, Orthology Assignments, and Domain Prediction at the Metagenomic Scale. Mol Biol Evol. 2021;38(12):5825–9. 34597405 10.1093/molbev/msab293 PMC8662613

[43] Wu T, Hu E, Xu S, Chen M, Guo P, Dai Z, Feng T, Zhou L, Tang W, Zhan L, et al. clusterProfiler 4.0: A universal enrichment tool for interpreting omics data. Innov (Camb). 2021;2(3):100141. 10.1016/j.xinn.2021.100141 PMC8454663 34557778

[44] Langfelder P, Horvath S. WGCNA: an R package for weighted correlation network analysis. BMC Bioinformatics. 2008;9:559. 19114008 10.1186/1471-2105-9-559 PMC2631488

[45] Yang Z, Cao Y, Shi Y, Qin F, Jiang C, Yang S. Genetic and molecular exploration of maize environmental stress resilience: Toward sustainable agriculture. Mol Plant. 2023;16(10):1496–517. 37464740 10.1016/j.molp.2023.07.005

[46] Masuka B, Araus JL, Das B, Sonder K, Cairns JE. Phenotyping for abiotic stress tolerance in maize. J Integr Plant Biol. 2012;54(4):238–49. 22443263 10.1111/j.1744-7909.2012.01118.x

[47] Li H, Wang J, Li M, Wu L, Rao W, Peng X, Jiang H. The ZmHSF08-ZmUGT92A1 module regulates heat tolerance by altering reactive oxygen species levels in maize. Crop J. 2024;12(5):1437–46.

[48] Zeng H, Dou D, Yang Y, Yan Y, Sun Y, Yang S, Yao W, Zhao S, Wang M, Liu Z et al. The ZmFKF1b-ZmDi19-5 Regulatory Module Coordinates Drought Tolerance and Flowering Time in Maize. Plant Biotechnol J. 2026;24(3):1044–60. 10.1111/pbi.70404 PMC12946474 41060050

[49] Gu L, Hou Y, Sun Y, Chen X, Wang G, Wang H, Zhu B, Du X. The maize WRKY transcription factor ZmWRKY64 confers cadmium tolerance in Arabidopsis and maize (Zea mays L). Plant Cell Rep. 2024;43(2):44. 38246890 10.1007/s00299-023-03112-8

[50] Sharma SS, Dietz K-J. The relationship between metal toxicity and cellular redox imbalance. Trends Plant Sci. 2009;14(1):43–50. 19070530 10.1016/j.tplants.2008.10.007

[51] DalCorso G, Manara A, Furini A. An overview of heavy metal challenge in plants: from roots to shoots. Metallomics. 2013;5(9):1117–32. 23739766 10.1039/c3mt00038a

[52] Yang X, Ren J, Yang W, Xue J, Gao Z, Yang Z. Hydrogen sulfide alleviates chromium toxicity by promoting chromium sequestration and re-establishing redox homeostasis in Zea mays L. Environ Pollut. 2023;332:121958. 37286026 10.1016/j.envpol.2023.121958

